# Adaptive Bayesian Clinical Trials: The Past, Present, and Future of Clinical Research

**DOI:** 10.3390/jcm14155267

**Published:** 2025-07-25

**Authors:** Donald A. Berry

**Affiliations:** 1 Department of Biostatistics, The University of Texas M.D. Anderson Cancer Center, Houston, TX 77030, USA; don@berryconsultants.net; 2 Berry Consultants, LLC, Austin, TX 78746, USA

**Keywords:** bandit problems, simulating clinical trials, complex innovative designs, artificial intelligence, Bayesian predictive probabilities, predicting clinical trial outcomes, interim analyses, continually monitoring clinical trials

## Abstract

**Background/Objectives:** Decision-analytic Bayesian approaches are ideally suited for designing clinical trials. They have been used increasingly over the last 30 years in developing medical devices and drugs. A prototype trial is a bandit problem in which treating participants is as important as treating patients in clinical practice after the trial. **Methods:** This article chronicles the use of the Bayesian approach in clinical trials motivated by bandit problems. It provides a comprehensive historical and practical review of Bayesian adaptive trials, with a focus on bandit-inspired designs. **Results**: The 20th century saw advances in Bayesian methodology involving computer simulation. In the 21st century, methods motivated by bandit problems have been applied in designing scores of actual clinical trials. Fifteen such trials are described. By far the most important Bayesian contributions in clinical trials are the abilities to observe the accumulating results and to modify the future course of the trial on the basis of these observations. In the spirit of artificial intelligence, algorithms are programmed to learn the optimal treatment assignments over the remainder of the trial. **Conclusions**: Bayesian trials are still nascent and represent a small minority of clinical trials, but their existence is changing the way investigators, regulators, and government and industry sponsors view innovation in clinical trials.

## 1. Introduction

Bayesian approaches are used increasingly in designing clinical trials. They are enabling revolutionary modifications in the building and running of clinical trials. Understanding today’s current status of clinical research requires understanding some of the differences in statistical philosophy and in some of the evolution of the Bayesian approach.

Understanding the future of clinical trials requires learning some of the history of how we got to where we are today. The road has been long and winding. In this article, I will describe some of my role in the journey. It includes some successes and some bumps and hazards encountered along the way—many of which still exist in some quarters. The most important aspect of the story is the U.S. FDA’s critical role in facilitating innovation.

## 2. Frequentist Statistics Versus Bayesian Decision Analysis

My definition of statistics is decision-making under uncertainty. Designing a clinical trial is a decision problem. The trial’s goal determines its optimal design. The traditional goal of clinical trials is science; namely, getting information about a therapy’s safety and efficacy that applies to the larger patient population. This goal is codified in the Belmont Report, which is the widely accepted bible of clinical research ethics [1]. This 45-year-old document makes clear that medical research (that is, clinical trials) is distinct from medical practice (that is, treating patients). To wit: “The purpose of medical or behavioral practice is to provide diagnosis, preventive treatment or therapy to particular individuals. By contrast, the term ‘research’ designates an activity designed to test an hypothesis, permit conclusions to be drawn, and thereby to develop or contribute to generalizable knowledge (expressed, for example, in theories, principles, and statements of relationships).” [1].

A fundamental principle of testing hypotheses is controlling the type I error rate. This is the probability the trial will conclude an investigational therapy is effective under the assumption that the therapy is not effective (if you prefer addressing the probability that the therapy is effective, you are not alone, and you are ready for exposures to the Bayesian approach). When the Belmont Report was published, the relevance of Bayesian decision-theoretic ideas in clinical research was not appreciated by ethicists and clinical trialists. The Belmont Report is outdated. It should be revised to recognize that clinical trials can simultaneously achieve both good science and good treatment for trial participants. The experience of I-SPY 2 proves this statement.

A trial’s type I error rate is prospective. It is a characteristic of the trial’s design and not of the trial’s outcome. The analogue measure in terms of a trial’s outcome is the *p*-value.

Testing hypotheses and contributing to generalizable knowledge are not the best goals for clinical trials. They are relevant but they are at least one level removed from the most appropriate goal of clinical research, with is treating patients effectively. Learning about treatment effectiveness is good, but it has a cost, and I do not mean a monetary cost. The relevant cost is in terms of potentially ineffective therapy of patients in the trial. Effective treatment of a patient in a clinical trial is as important as that of a patient who presents after the trial. This is so even though the latter patient has the obvious advantage of availability of information gleaned from the outcomes of the former patient.

Clinical trial designs should depend on the disease. In particular, they should depend on the size of the patient population having the disease, now and in the future. As extreme examples, consider clinical trials investigating potential cures in coronary artery disease (CAD) versus Tay–Sachs disease (TSD). CAD kills about 380,000 (mostly elderly) people per year in the United States, while TSD is a fatal autosomal recessive genetic disease that affects fewer than 50 newborns per year in the United States.

Testing hypotheses in developing generalizable knowledge may be reasonable for CAD. Indeed, one might argue that randomizing hundreds or even thousands of CAD patients in a doubly blinded trial while controlling type I error is an approximate solution to the decision analysis problem. For TSD, on the other hand, randomizing in a clinical trial would be not only logistically difficult but also unethical. For rare diseases quite generally, randomizing hundreds of patients in a clinical trial is impossible and controlling type I error is irrelevant.

A better strategy for both CAD and TSD—and for all diseases with prevalence in between—is to use an adaptive design that recognizes the pros and cons of increasing the sample size given the prevalence of the disease. Additionally, the design should utilize information accumulating in the trial for treating patients to come, those in and those outside the trial [2,3,4,5,6,7].

Accumulating information in all cases includes longitudinal effects on patients other than death that are known to be or may be related to the disease. In CAD, a therapy that prolongs survival would be expected to decrease chest pain, dyspnea, fatigue, etc. Information about these symptoms, correlations with death, and correlations with each other by each therapy should be used in understanding the effects of the therapy. Symptoms of TSD involve diminution of the senses, including the ability to move. Again, a therapy that prolongs survival would be expected to have an effect on the symptoms of the disease, but not always.

Appropriate adaptations for CAD trials include re-estimating sample sizes and adjusting randomization probabilities. Such adjustments include the possibility of setting an arm’s randomization probability to 0, which means pausing accrual in the arm in question and possibly permanently stopping the arm’s accrual. For TSD, the optimal adaptive design is simple and, I hope, obvious. Namely, when a new investigational therapy is proposed, assign it to available patients until the death rate shows that it is not an improvement compared with current standard of care. The current “standard of care” is updated over time, including based on results of other therapies being considered in the trial.

## 3. Bayesian Bandit Problems

When I first learned about fixed randomization in clinical trials, I thought that equipoise may exist at the start of a trial and so rolling a *k*-sided die to determine therapy for the first few patients might be reasonable. However, as results from patients treated earlier in the trial become available, some therapies will be performing better than others. It seems unethical to continue to randomize patients to therapies that are doing poorly, or so I thought. I was surprised to learn that trialists did exactly that. They avoided an ethical dilemma by blinding themselves to patient outcomes. Blinding oneself to avoid information that may have ethical implications is itself unethical!

My PhD dissertation at Yale University in 1971 was the foundation for developing my view of clinical trial design [8]. I took a Bayesian approach because that was and is the only practical way to update information that accrues during a clinical trial, based on accumulating data. I was particularly intrigued by a problem in decision theory that came to be known as a multiarmed bandit [9]. I regarded two-armed and multiarmed bandits as ideal models for clinical trials, and indeed for other types of sequential choice problems. I still do. The real problem was, as I will describe here, clinical researchers strongly disagreed with me.

As a model for clinical trials, I considered a population of patients with a particular disease that would be treated with one of *k* available therapies (arms). Let *n* denote the size of this population. Patients in the population arrive one at a time. The simplest version of a *k*-armed bandit is when *k* = 2 and patient outcomes are dichotomous (success/failure) and become known before the next patient arrives for treatment. The goal is maximizing the expected number (or, equivalently, expected proportion) of successes in the *n* patients.

A more general problem, and one that better fits most clinical trials, is to view *n* − 1 as an adaptive trial sample size and have *N*, say, be the full population size, including those who will present with the disease or condition after the trial. Anscombe [10] and Colton [11] independently introduced this notion of “patient horizon.” Then, in the mathematics the *n*th “patient” represents the full set of the remaining *N* − *n* + 1 patients. Consistent with the bandit-problem goal, the remaining population of *N* − *n* + 1 patients would be treated with the therapy that performed better during the clinical trial involving *n* − 1 patients [12]. As another generalization, the full population size *N* will likely be unknown and therefore would have a probability distribution [13].

A bandit strategy is a scheme for deciding which therapy to use for the next patient for every possible sequence of treatments and outcomes up to that time. In 1952, the famous (non-Bayesian) mathematician Herbert Robbins proposed a simple bandit strategy called “Win–Stay, Lose–Shift.” Following this strategy, randomize treatment arm for the first patient. Then whenever a patient’s outcome is successful, use the same arm for the next patient, and if a patient’s outcome was a failure, then switch to the other arm [14]. This is a strategy because it prescribes the next therapy prospectively for every possible set of outcomes. Robbins showed that this simple strategy was better than any strategy that ignored the accumulating data in assigning treatments, which includes randomization. However, he recognized that there were better strategies than Win–Stay, Lose–Shift. Robbins’ initial research led many authors (including Robbins himself) to propose strategies that improved on previously proposed strategies. Some of the proposed strategies demonstrated improvements only in a restricted set of parameters, *p*_1_ and *p*_2_, the true probabilities of success for the two arms. However, no research came close to proposing a strategy that was best for all *p*_1_, *p*_2_, and *N*—and for good reason.

The Robbins-led research approach highlights a major difference in the way Bayesians and frequentists approach decision problems. Frequentists can propose strategies and evaluate them as a function of the unknown *p*_1_ and *p*_2_. Parameters in the frequentist approach are regarded to be fixed but unknown. In the Bayesian approach, all unknowns have probability distributions. This allows Bayesians to go beyond frequentists by calculating the value of a strategy unconditionally on *p*_1_ and *p*_2_. Namely, Bayesians integrate over *p*_1_ and *p*_2_ with respect to their probability distribution. A probability distribution for *p*_1_ and *p*_2_ applies at the start of a trial—it is the so-called prior distribution. Then, during the trial, the current (or posterior) probability distribution is the one updated by the data observed up to that time.

A major advantage of a Bayesian approach is the ability to consider goals such as maximizing the expected number of successful outcomes in a clinical trial without having to condition on *p*_1_ and *p*_2_. However, this advantage has a cost. Bayesians must assess a prior distribution for *p*_1_ and *p*_2_ and they must be able to find the updated probability distributions of the unknown parameters at future decision epochs. These distributions reflect the uncertainty present at the time. The distributions depend on what statisticians call “sufficient statistics,” which in the case at hand are the numbers of successes and failures so far observed on the two arms. Win–Stay, Lose–Shift and its generalizations are not based on sufficient statistics.

When seeking good strategies, frequentists face the following conundrum. They can calculate the expected number of successes for fixed *p*_1_ and *p*_2_ for any strategy—in theory, at least. In one sense, finding an optimal strategy is easy for frequentists. Namely, since *p*_1_ and *p*_2_ are fixed, if *p*_1_ > *p*_2_ then always use arm 1 and if *p*_2_ > *p*_1_ then always use arm 2. However, this is a dead end. In any actual setting, *p*_1_ and *p*_2_—and parameters more generally—are unknown. It is only during the trial that information will be forthcoming to reveal which *p*_i_ is likely to be larger. Frequentists are hampered by not being able to view unknown quantities as random variables. They do not have a mechanism for updating the available information about unknown parameters based on accumulating data.

Finding an optimal strategy in the Bayesian approach requires considering the outcome of the current patient but also getting information about the available therapies that could help in selecting therapies for future patients. These two desiderata are sometimes called “earn and learn” or “exploit and explore.” They are inseparable in bandit problems. Earning and learning are frequently conflicting. By this, I mean that one therapy may be best for earning—treating the current patient as effectively as possible—while a different therapy is best for learning in the sense of getting information that will help in treating future patients.

Finding an optimal strategy is difficult because these conflicting desiderata cannot be separated. Bandits are in a class of problems computer scientists call *N-P* hard [15]. However, optimal strategies can be found numerically for any particular Bayesian multiarmed bandit using dynamic programming (backward induction). Computer storage requirements increase as the cube of *n* [8,9,16]. Start the backward induction by specifying all possible probability distributions of *p*_1_ and *p*_2_ after the first *n* − 1 patients have been treated. The decision problem for patient *n* is easy because the only goal that now counts is earning; there is no utility for anything learned after the last patient is treated. Namely, for patient *n* select the arm with the higher probability of success; the probability of success on arm *i* is the expected value (mean) of *p*_i_. Work backwards starting from *n* − 1. Keep track of the expected number of future successes for all possible outcomes. Mark the optimal next selection as the one that maximizes the expected number of successes if you use arm *i* and then follow an optimal continuation depending on whether the patient’s result was a success or failure. You can do this because you have written down all the possibilities when you have one more observation on either arm, and you have also written down the maximal expected number of successes in the future for every possibility with one more observation on either arm. Many references are available that give the details in backward induction for bandit problems [8,9,16].

I will find the optimal strategy in a very simple example. I can take a forward approach because the example is so simple. One of my goals is to show how updating occurs with every outcome in the Bayesian approach. Another is to illustrate how Bayesian probabilities of future outcomes (predictive probabilities) are useful in a clinical trial. Still another is to demonstrate the inseparable nature of earn and learn. This simple example shows why it is better to adapt. It provides very simple calculations showing that the expected number of successful treatments is larger for a bandit strategy than for the conventional equally randomized (non-adaptive) trial.

**Figure 1:**
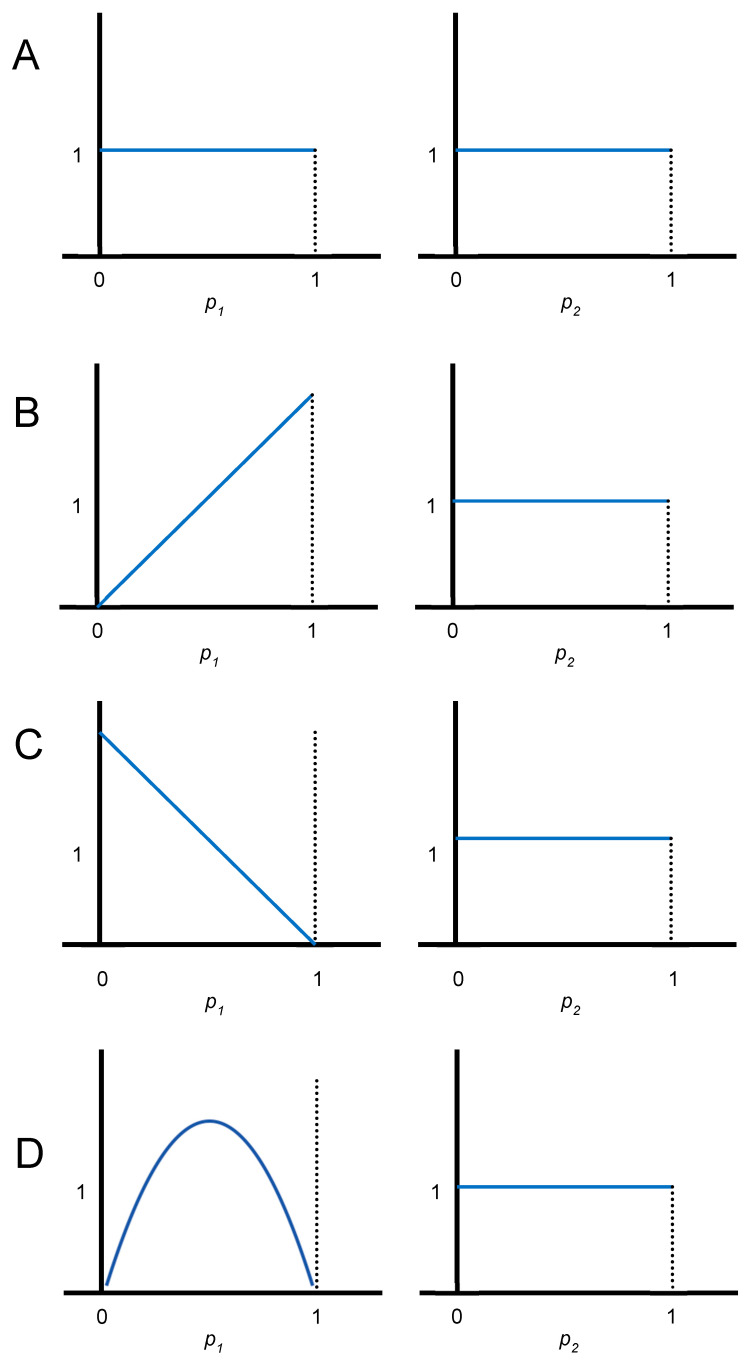
(**A**) Prior probability distributions of success rates *p*_1_ and *p*_2_. The mean is 1/2 for both arms. Since the two distributions are the same, the arms are exchangeable and therefore both arms are optimal choices for patient #1. (**B**) Probability distributions of success rates *p*_1_ and *p*_2_ after a success on arm 1. The current mean for *p*_1_ is 2/3. With 3 more patients to go, the optimal choice for patient #2 is arm 1, staying with a winner. (**C**) Probability distributions of success rates *p*_1_ and *p*_2_ after an initial failure on arm 1. The mean of *p*_1_ is now 1/3. With 3 more patients to go, the optimal choice for patient #2 is arm 2. (**D**) Probability distributions of success rates *p*_1_ and *p*_2_ after a success and a failure (or a failure and then a success) on arm 1. The mean for *p*_1_ is again 1/2, the same as for *p*_2_. If patient #3 were the last patient to be treated, then both arms would be optimal. However, there is more information to be gained using arm 2 for patient #3 because of patient #4. Therefore, the only optimal choice for patient #3 is arm 2.

Assume independent uniform prior distributions for the two parameters *p*_1_ and *p*_2_. These are shown in panel A of Figure 1. The prior expected value of both *p*_i_’s is 1/2. the probability of success for any patient treated with an arm in the clinical trial (including the first patient) is the expected value of the current distribution of the corresponding *p*_i_. Therefore, in the absence of information about the two success rates, assigning any patient to either arm 1 or arm 2 yields a success for that patient with probability 1/2. In addition, randomizing the *n* patients (or using either only arm 1 or only arm 2 for the *n* patients) while ignoring accumulating evidence in the trial would give an expected overall success proportion of 1/2, regardless of the randomization probabilities.

The optimal bandit strategy is the trade-off between treating the current patient effectively and gaining information to help treating later patients. Consider the simple case of *n* = 4, with patients #1 through #4 to be treated in numerical order. The goal is to maximize the expected number (or equivalently, expected proportion) of successes for these four patients. The information from the previously treated patients is used to update the probability distributions of *p*_1_ and *p*_2_ using Bayes rule, with each treatment assignment made to maximize the expected number of successes in the remainder of the trial. Despite the tiny population size of four, I will show that the expected proportion of successes is measurably greater than 1/2, which is achievable by any randomizing strategy, or by any other strategy that ignores the accumulating data in the trial.

Since the prior information is symmetric in the two arms (panel A of Figure 1), both arms are optimal for patient #1 (when the arms are exchangeable (that is, the joint distribution of (*p*_1_, *p*_2_) is the same as the joint distribution of (*p*_2_, *p*_1_)) is one of the few circumstances for which randomization is optimal in a bandit problem). Suppose you select arm 1 for patient #1 and observe a success. The updated probability distribution for *p*_1_ is shown in panel B of Figure 1, with the distribution for *p*_2_ remaining unchanged. Now the only optimal continuation is to stay with arm 1 for patient #2. This is despite the fact that the information content of assigning arm 2 is greater than that for arm 1 (staying with a winner is a characteristic of optimal bandit strategies more generally, provided that the arms are independent [8]).

The other possible outcome for patient #1 is that arm 1 failed (panel C of Figure 1). In that case, arm 2 is the optimal assignment for patient #2 (however, switching from a loser is not always optimal).

The only interesting continuation after patient #2 is when arm 1 succeeds for patient #1 and then fails for patient #2. The current distributions of *p*_1_ and *p*_2_ are shown in panel D. Even though the expected values of the two distributions in panel D are the same, with both equal to 1/2, arm 2 is the uniquely optimal assignment for patient #3. The intuition is that even though the probabilities of success for patient #3 are the same, arm 2 provides more information relevant for improving the success rates for later patients, which in this simple example is limited to patient #4. As generally true, the last patient, #4, should receive the arm that has the larger mean at that time.

The success rate for the optimal strategy in this example is 5/9 = 0.556. This is the average over the four patients. Order matters. The four patients have respective success rates of 1/2, 7/12, 5/9, and 7/12, which in decimal form are 0.500, 0.583, 0.556, and 0.583. The overall success rate for an optimal strategy increases with *n*. This is a reflection of the meta-principle that it is always better to delay getting a disease (the success rate tends to increase as well with patient number, but as the simple example shows, the increase is not necessarily monotone). For *n* = 100, the overall success rate is 0.64918, as I advertised in an article I wrote in 1973 as part of my efforts to interest clinical trialists in using Bayesian adaptive trials [9]. This rate for *n* = 100 is close to the asymptotic (*n* → ∞) success rate of 2/3. Its calculation is the expected value of max{*p*_1_, *p*_2_} based on the prior distributions (panel A of Figure 1). An interpretation of the incremental success rate from *n* = 1 to *n* = ∞ (which is 2/3 − 1/2 = 1/6) is what decision analysts call the expected value of perfect information (EVPI) [17]. It is the expected value of learning the true values of *p*_1_ and *p*_2_ at the start of the clinical trial because you would then use the arm with the larger *p_i_* for all patients.

I published my dissertation in the mathematical statistics literature [8]. I continued to publish on bandit problems, partly to encourage clinical trialists to take a bandit approach. On that score I failed miserably. I was criticized by biostatisticians and clinical trialists for being naïve regarding clinical trials. However, their criticism was appropriate. I was naïve. I learned that I had to become an expert in designing traditional clinical trials and respected in their world in order to change them.

## 4. Duke University, CALGB, and Clinical Trials in Breast Cancer

In the 1970s and 1980s, I worked to achieve credibility in the traditional clinical trial community. An article in *Science* by Jennifer Couzin chronicles the path I followed: “A big break for Berry came in 1990, when he was invited to join Cancer and Leukemia Group B (CALGB).” [18]. At that time, I had moved from the University of Minnesota to professor in Duke’s Institute of Statistics and Decision Sciences. It happened that Duke housed the Statistical Center for the CALGB, a national oncology group that designed and ran national clinical trials in the most prevalent cancers. “Berry would be the lead statistician for CALGB’s breast cancer studies. He was not greeted warmly.” [18].

Couzin continues: “‘I objected rather strenuously,’ recalls I. Craig Henderson, a breast oncologist at the University of California, San Francisco, who had heard that Bayesians were ‘loosey-goosey’ in adhering to the rules. Henderson subsequently had a change of heart: Last year (2003), he was the first in a string of authors on one of the largest breast cancer studies Berry has designed, with more than 3000 women. Its factorial design revealed that adding the drug paclitaxel (Taxol) to standard chemotherapy is beneficial, and that high doses of doxorubicin (Adriamycin), one of the most toxic chemotherapy agents, don’t fight cancer any more effectively than lower doses. This came as a great surprise, and some criticized the study for its unusual methodology.” [18].

Craig Henderson became one of my best friends and an avid supporter of Bayesian approaches, as well as a supporter of my penchant for factorial designs [19,20,21,22,23,24].

Couzin continued: “Despite Berry’s relentless efforts to convert Bayesian nonbelievers in CALGB, the group has yet to conduct a fully Bayesian study. But CALGB and other cooperative groups are adopting factorial designs to answer more questions, more quickly.” [18].

As an update to Couzin’s comment in 2004 about not having conducted a “fully Bayesian study,” I had actually designed such a study, CALGB 49907. See below. However, in 2004 the trial was still enrolling patients.

I moved to the University of Texas M.D. Anderson Cancer Center in 1999 to found a Department of Biostatistics and eventually a Division of Quantitative Sciences. I used the Bayesian approach at M.D. Anderson when designing early-phase clinical trials, and I encouraged other members of the department to do the same. This experience was chronicled by Biswas et al. [25]. The Biswas article prompted the editorialist to comment, “While there are certainly some at other centers, the bulk of applied Bayesian clinical trial design in this country is largely confined to a single zip code.” [26]. This statement is no longer accurate. Bayesian designs have spread well beyond M.D. Anderson, and also well beyond the United States.

Nonetheless, I had to face up to the important issue of randomization. Randomization in the clinical trial community was and is sacrosanct. It is the widely respected gold standard of medical research. In those early trials at M.D. Anderson, I used a generalization of a particular adaptive Bayesian bandit strategy for a clinical trial that had been proposed in 1933 by W.R. Thompson [16,27]. Thompson considered the case *k* = 2, though his proposal easily generalizes to *k* > 2. Actually, his proposal is much more useful and effective when *k* is larger, such as in platform trials. Thompson proposed that the next patient should to be treated with arm 1 with the current Bayesian probability that arm 1 is better than arm 2. Thompson remarked that “even though [this strategy is] not the best possible, it seems apparent that a considerable saving of individuals otherwise sacrificed to the inferior treatment might be effected.” [27].

Thompson’s work predated the Bayesian/frequentist feuding that occurred in the middle of the 20th century. However, it is not clear whether he regarded himself to be a Bayesian. He does say “it must be decided whether these requirements are met or not, and whether we may apply the well-known Principle of Bayes to convert the problem to the form of Section 2. Statistical criteria are often employed, however, in situations in which certain deviations from the conditions required in their development can be tolerated, when a better procedure is not available.” [27].

Fast forward to the 1970s, when the Bayesian/frequentist feud was ripe. The great statistician Herman Chernoff had made substantial contributions to statistical theory and practice, including in bandit-like problems. He said to me, “I’m not a Bayesian.” [28]. He proceeded to give essentially the same explanation as Thompson’s. He said he used the Bayesian approach because Bayes rule was the only available tool that would do what he needed to do, which was to quantify and update his knowledge after each patient’s outcome.

The person widely credited for introducing randomization into clinical trials was Sir Austin Bradford Hill. Hill designed and helped conduct the world’s first randomized clinical trial. It evaluated streptomycin in tuberculosis [29]. Interestingly, although Thompson’s proposal was theoretical and he never used it in a clinical trial, it predated Hill’s publication by 15 years. Additionally, Thompson’s adaptive randomization proposal is more flexible and arguably more useful than Hill’s because it allows the randomization probabilities to vary over time depending on the accumulating results in the trial and on prior information available in advance of the trial.

Thompson was driven by the ethical concerns regarding “sacrificing patients” to the inferior treatment. It is ironic that I have been criticized for being unethical because I have adapted Thompson’s strategy in actual clinical trials [30,31] (see my respective responses [32,33]). In a more recent article entitled *“*Adaptive clinical trials: A partial remedy for the therapeutic misconception?”, Meurer et al. [4] argue from the same ethical perspective as Thompson. The therapeutic misconception is that “Some trial participants and family members believe that the goal of a clinical trial is to improve their outcomes.” Moreover, they claim that adaptive randomization a la Thompson (also called response-adaptive randomization) is a route for merging clinical research and clinical practice. Such a merger is inevitable, and it is consistent with my recommendation for revising the Belmont Report. The following is the conclusion of the Meurer article:

“By using adaptive clinical trial designs that incorporate response-adaptive randomization, we can realize the scientific benefits of traditional randomization while offering trial participants a greater chance of receiving superior treatments. After years of training clinical researchers to inform patients that a clinical trial will probably not benefit them directly and having that message lost on many clinical trial volunteers, the research community should start ensuring that clinical trial goals include improving the expected outcomes of the trial participants. Doing so will help to more closely align the aims of science and the desires of research participants.”

[2]

**Figure 2:**
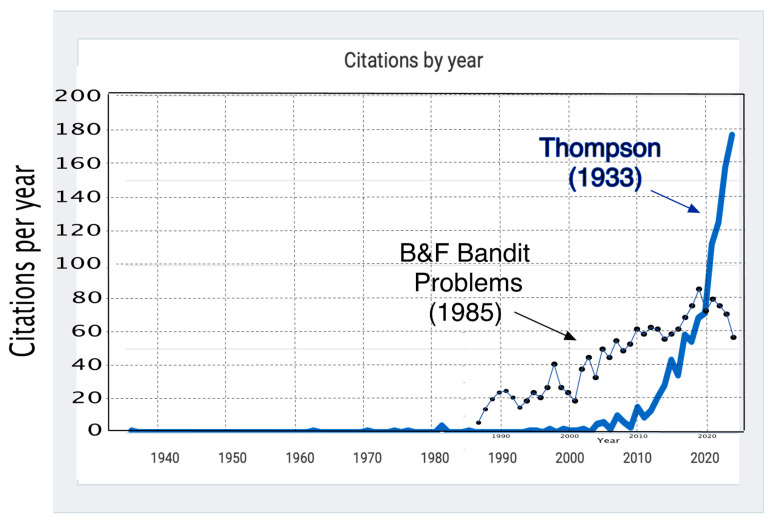
Numbers of citations of references B&F [16] and WRT [27] since their respective publications in 1985 and 1933. Total citations for B&F and WRT are 1767 and 1087. WRT was one of the entries in the annotated bibliography of B&F and so the tiny blip at 1985 in the WRT plot contains the citation of WRT by B&F. The continued increase in rate of citations after 40 and 90 years since publication is not typical in science or medicine. The exponential-like explosion of citations to WRT after 70 years of smatterings may be unique. The irony is that the last 9 pages of the 10 pages of WRT describe a method for computing the probability that one random variable is greater than another. Since that probability involves convolutions of incomplete beta functions, its calculation was incredibly tedious for the adding machines of 1933. Those 9 pages became unnecessary with the advent of computers. As B&F pointed out in their annotated bibliography, the only interesting page of WRT in the modern world is the first one [16].

The preface of the book *Bandit Problems* includes the following: “Throughout the book the authors take pains to teach the material rather than simply giving theorems and proofs. *Bandit Problems* should therefore be accessible to a wide audience including not only graduate students, professionals and academics in statistics, but also in mathematics, biomedicine, engineering, economics, management science, operations research and psychology.” [16]. If the increased application of bandit strategies in these fields is any indication, the book may have been partly responsible. In any case, the bandit literature continues to increase, along with citations to the book. This is despite the fact that the book is 40 years old while the applications are au courant (see Figure 2). These recent citations are from all areas of application listed in the book’s preface. Of special significance in terms of publication volume are applications in operations research by search engines. The bandit “earn/learn” conundrum involves the choice of ads to display to users; the search engine earns when a user clicks on an ad. Figure 2 shows an even more explosive number of recent citations for Thompson’s 1933 [27] article on adaptive randomization. Again, the areas of application for adaptive randomization are broad, but with much of the explosion in search-engine research.

## 5. Adaptive Bayesian Clinical Trials in Practice

**Figure 3:**
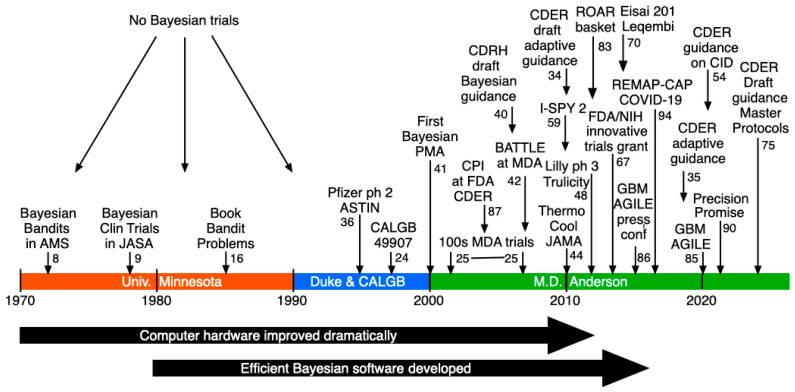
Timeline of Bayesian adaptive clinical trials. Donald Berry at the University of Minnesota, then Duke University, plus the Cancer & Leukemia Group B (CALGB), and then the University of Texas M.D. Anderson Cancer Center (MDACC) plus Berry Consultants. Arrows on clinical trials indicate time of the initial design. Numbers on the arrows indicate the relevant references.

Figure 3 is a pictorial summary of some of my experience regarding adaptive Bayesian clinical trials. The list of trials is partial. The only time when the list is complete is prior to 1995, when there were no Bayesian clinical trials. In this Section 1 will provide some details and references for most of the trials cited in Figure 3. The figure’s approximate chronology is based on the year in which the respective trial’s design was initiated; neighboring intervals of patient accrual and follow-up are frequently overlapping. The trials form a chain with links contributing innovations and improvements that are available for use by subsequent trials. Figure 3 includes FDA guidances interlaced within the chain of trials. The trials clearly benefited from these guidances and some of the guidances benefited from the trials. The numerical label on arrows in Figure 3 are principal references for the items indicated.

There was a sea change in the FDA’s attitude toward the Bayesian approach in clinical trials between their draft adaptive design guidance in 2010 and their finalized guidance nine years later. In 2010, this reflected their understanding of simulation: “Some modeling and simulation strategies lend themselves to a Bayesian approach that might be useful.” However, they continued thusly: “Using simulations to demonstrate control of the Type I error rate, however, is controversial and not fully understood.” [34]. Nine years later, they were completely on board: “The guidance also advises sponsors on the types of information to submit to facilitate FDA evaluation of clinical trials with adaptive designs, including Bayesian adaptive and complex trials that rely on computer simulations for their design.” [35].

Moreover, the FDA’s 2019 guidance was helpful in other ways. For example, regulators are concerned about controlling type I error rate throughout the “null hypothesis space.” There are ancillary aspects (or nuisance parameters) that affect type I error. For example, simulations may not have considered accrual rates that actually occurred in the trial. I visited the FDA in 2015 and presented them with a proposal to handle such possibilities *after the trial*. I called it “post-simulation.” Their 2019 guidance addresses this possibility in detail, but not using that name. For example, “However, with any approach, the evaluation at the end of the trial should consider whether the statistical inference is appropriate and the conclusions are justified in light of the accumulated information about the nuisance parameters. In the example, if the observed placebo mortality rate was unexpectedly 50 percent, additional simulations would be required.” [35].

1. **CALGB (Cancer & Leukemia Group B) 49907**

CALGB 49907 was a fully Bayesian clinical trial addressing whether capecitabine (Xeloda^®^) is non-inferior to standard chemotherapy in non-metastatic breast cancer patients who were at least 65 years old at diagnosis. I began designing the trial in 1999. The first draft of the design was traditional. I modified it several times over the next 2+ years before the National Cancer Institute (NCI), which would be the trial’s sponsor, accepted it. The NCI insisted that a minimum of 1800 patients would be necessary to answer the trial’s scientific question: “Is capecitabine non-inferior to standard chemotherapy in this population?” I reported to the NCI that even though we would be accruing patients nationwide, in my experience it would take at least 15 years to accrue and follow 1800 patients in this elderly population. I suggested that by the time we answered the scientific question it would probably no longer be interesting. I got nowhere.

Finally, and out of frustration, I told the NCI we would satisfy their requirement of 1800 patients but only if the interim results indicated that 1800 patients would be necessary. We would accomplish this by periodically calculating Bayesian predictive probabilities that the currently enrolled patients would likely be sufficient for answering the question with further follow-up but with no further enrolment. We would start the sequence of analyses by making this calculation when the 600th patient enrolled. If the trial did not stop at 600 patients, then we would repeat the calculations after 900, 1200, and 1500 patients had been enrolled. The NCI accepted my proposal.

We published the results of the trial in 2009, at which time the CALGB had indeed conducted its first fully Bayesian study. Moreover, its publication was, I believe, the first fully Bayesian randomized trial published in the New England Journal of Medicine [24]. The prospective Bayesian design called for stopping accrual at any interim analysis if the predictive probability of getting a meaningful answer was at least 80%. Based on this criterion, the trial stopped enrollment at the first interim analysis with 633 patients enrolled. As the interim Bayesian calculations had predicted, additional follow-up of 16 months in the trial delivered a compellingly negative answer. Namely, chemotherapy cannot be replaced by capecitabine in elderly breast cancer patients. The Journal emphasized the fully Bayesian aspect of the trial’s design. For example, the abstract indicates that “A Bayesian statistical design was used with a range in sample size from 600 to 1800 patients.” [24].

2. **Pfizer adaptive dose-finding Acute Stroke Therapy by Inhibition of Neutrophils (ASTIN) trial**

In the early 1990s, neurologist Michael Krams at Pfizer attended a weeklong course I gave at Pfizer’s facilities. After the course he asked if I would design a Bayesian dose-finding phase 2 trial in acute ischemic stroke for an investigational drug called UK-279,276, a neutrophil inhibitory glycoprotein. The trial was ASTIN (Acute Stroke Therapy by Inhibition of Neutrophils) [7,36,37,38]. Its goals were to determine whether the drug was efficacious in stroke and, if so, to identify its best dose. This was defined to be the ED95, the dose that achieves at least 95% of the maximum efficacy of the drug across the dose spectrum. We used 15 doses of the drug from 10 to 120 mg plus placebo. A minimum of 20% of the patients would be randomly assigned to placebo, but more patients on placebo were allowed if that would be the most informative dose for finding the ED95. We knew that the algorithm would concentrate on only a small number of the doses, but we did not know which doses would attract its attention. The algorithm did not “know” either, not until “seeing” the accumulating trial results.

The primary efficacy endpoint of ASTIN was change from baseline to day-90 Scandinavian Stroke Scale (SSS). We built an algorithm that would analyze the updated results daily and determine the dose or doses to be used the next day. The algorithm smoothed estimates across doses using a Normal Dynamic Linear Model (NDLM) [39]. In addition, we used a longitudinal model to predict day-90 SSS for each patient using their weekly measurements of SSS for patients not yet having their 90-day visit [36].

The trial’s algorithm worked as planned perfectly [37]. It started out focusing on low doses and placebo. Observing what seemed to be no effect at the low doses, it kept increasing the range of doses investigated until it was concentrating only on the highest doses and placebo.

The algorithm had some administrative limitations. For example, it could not stop the trial for futility until 500 patients had 90-day SSS. By the time that the minimum number of completers had occurred, the trial had randomized 966 patients. The algorithm had long since given up finding an effective dose and stopped the trial because the sample size had reached its minimum. It would have been even smaller had the accrual rate been slower, but there was still a substantial saving compared with the maximum sample size of 1300.

3. **Pathbreaking Bayesian premarket approvals (PMAs) for medical devices**

The FDA’s Center for Devices and Radiological Health (CDRH) was early in addressing the use of the Bayesian approach in registration trials. In 1997 they contracted with me to write a white paper entitled “Using a Bayesian Approach in Medical Device Development.” The objective was to help them draft a Bayesian guidance, which was eventually issued in 2010 [40]. In those early days I designed several clinical trials evaluating spinal implants for Medtronic Sofamor Danek (Memphis, TN, USA), all of which took a Bayesian approach. These were non-inferiority trials. I described the statistical issues in a case study presented at a joint workshop of the FDA Centers for Drugs, Biologics, and Devices in May 2004 [41]. The focus of the workshop was Bayes in the regulatory setting [41]. The teachers were mostly from CDRH, or at least they were using CDRH examples. The students were mainly from CDER and CBER.

The two heroes in encouraging the Bayesian approach in medical device development at their respective institutions were Gregory Campbell, Director of Biostatistics at FDA’s CDRH, and Bailey Lipscomb of Sofamor Danek.

The principal innovation (besides taking the Bayesian approach) in early device trials that I designed was modeling the relationships between early and late endpoints, and then using Bayesian predictions for the late endpoints of those patients who have experienced the early endpoint but not yet reached the late endpoint. The concept is the same as predicting day-90 SSS from earlier visits in the ASTIN trial. If the results accruing in the trial showed a correlation, then there would be a substantial savings in trial duration, and possibly in sample size as well. If the results are uncorrelated, then the algorithm learns that they are uncorrelated and nothing is gained, but also nothing is lost.

In particular, the CDRH’s required follow-up time for spinal implants was (and still is) 24 months. The good news is that the primary measurements were available on the patients at 1.5, 3, 6, and 12 months. The following is the conclusion of our article in *Clinical Trials* mentioned above: “this case study shows how a Bayesian approach to clinical trial design allows for modeling early vs late endpoints, for predicting future observations, and, usually, for obtaining better answers more quickly.” [41].

4. **BATTLE trial at University of Texas M.D. Anderson Cancer Center**

The BATTLE Bayesian adaptive platform trial cited in Figure 3 was a precedent for I-SPY 2 [42]. BATTLE was designed by statistician Jack Lee of M.D. Anderson (Houston, TX, USA). The trial accrued 341 non-small cell lung cancer (NSCLC) patients from 2006 to 2009. It was the first master protocol conducted in oncology and perhaps more generally. The term “master protocol” was used to describe BATTLE. The term “platform trial” was not introduced into the taxonomy of clinical trials until later. I coined the term to describe I-SPY 2 [43]). BATTLE was also a basket trial in the sense that the NSCLC patient population was partitioned into five genomic biomarker-defined subtypes, with the therapies being evaluated within those subtypes. Like many of the contemporary Bayesian trials at M.D. Anderson, BATTLE utilized response-adaptive randomization [25,27].

5. **ThermoCool AF in JAMA, ablation catheter for atrial fibrillation**

NAVISTAR^®^ThermoCool^®^ is a BioSense Webster (Irvine, CA, USA) ablation catheter used for treating atrial fibrillation. It was compared with antiarrhythmic drugs in a pivotal clinical trial called ThermoCool AF (clinicaltrials.gov Identifier: NCT00116428) [44]. The trial started to accrue patients in 2004. However, patient accrual was much slower than expected and the sponsor was concerned. When they approached Berry Consultants, we recommended changing to a Bayesian design that would stop the trial for success with minimal sample size necessary for approval. We kept the original design of ThermoCool AF to the extent possible, except that we built Bayesian interim analyses into the design. These were based on the predictive probability of the final result of the trial when the currently randomized patients had long-term outcomes.

This change required several months of negotiations with the FDA’s CDRH while trial accrual continued. The first preplanned interim analysis was conducted after accruing 150 patients (out of a planned maximum of 230). Accrual to the trial was to stop (although follow-up would continue) if the predictive probability of statistical success with additional follow-up was greater than 98%. Moreover, if the predictive probability of success was greater than the protocol-specified threshold of 99% then this would lead to an immediate claim of study success, including stopping the trial and announcing the results. At this first interim analysis the predictive probability of long-term success was 99.9%, so the trial stopped and the results were submitted to the FDA. I called it a Goldilocks trial when presenting to the FDA’s advisory committee meeting, with sample size neither too small nor too big but just right.

The catheter was approved by the FDA in 2008 on the basis of ThermoCool AF. We submitted the results of the trial for publication to JAMA in 2009 [44]. My exchange with the JAMA Editor during their reviewing process was revealing. It demonstrated the prevailing attitude toward the Bayesian approach. That attitude persists among some researchers. An unfortunate example is the ROAR trial—see below.

The following was one of the editor’s comments to us regarding publishing the results of the ThermoCool AF study in JAMA:

“The main outcome is defined as ‘time to protocol-defined failure’. Such an analysis is generally a Kaplan–Meier analysis, with the association expressed as a hazard ratio. However, in the Abstract Results and in the text Results, primary results are presented as probability of success (which is based on a fixed timepoint of measuring success). K-M results are only in the text and figures and are secondary. It seems that the primary results in both the Abstract and text should be presented as hazard ratios with confidence intervals.”

This was my response to his comment:

“We appreciate the issue and have made changes to accommodate as well as we can. We feel that it is important to adhere to the general principle of following the protocol in design and analysis. This was a registration trial in which we worked closely with the FDA to build an efficient design based on Bayesian predictive probabilities. Stopping the trial was based on these probabilities and the primary analysis was specifically defined to be Bayesian (while incorporating an appropriate penalty for interim analyses). In the revised manuscript we have indicated that the prospective design and analysis were Bayesian, and we give the final Bayesian probabilities consistent with the protocol. However, after we give the prospectively defined analyses, all remaining analyses and measures of inference are conventional *P*-values and confidence intervals.”

In the 15 years following this episode, JAMA became Bayesian-friendly. As an example, JAMA published the aforementioned Meurer editorial [4]. I too have changed since my response to the Editor, particularly as regards the use of frequentist measures in reporting Bayesian trials. If forced to use frequentist measures, I caveat such use with the following in the article’s Methods section: “All *p*-values and confidence intervals in this article are descriptive and have no inferential content.” [45].

6. **First CDER approval of Bayesian NDA (New Drug Application): Pravigard Pac by Bristol Myers Squibb (BMS)**

BMS’s Pravastatin (Pravachol^®^, Bristol Myers Squibb, Princeton, NJ, USA) was an early statin. It was first approved by the FDA in 1991. It reduces the production of cholesterol in the liver, leading to a decrease in the levels of low-density lipoprotein (LDL) cholesterol, also known as bad cholesterol. Its indication is for lowering the risk of death due to heart disease and lowering the risk of heart attack. BMS’s Pravigard Pac combines pravastatin with aspirin. Pravigard Pac was approved by the FDA in 2003 to treat people with coronary heart disease and high cholesterol to lower the risk of stroke, heart attack, or other heart complications [46]. The basis for the approval was a Bayesian meta-analysis of five secondary prevention trials in which pravastatin was randomized but aspirin was not. All the other applications in this article involve designing Bayesian trials. We designed the meta-analysis, but that is different from designing a clinical trial. The reason I am citing Pravigard Pac in this article and giving it a slot in Figure 3 is that I believe that it was the first approval by the FDA’s Center for Drug Evaluation and Research (CDER) of an NDA that took the Bayesian approach as regards efficacy.

The fact that BMS’s statistical approach was not traditional seemed not to matter to the FDA. Neither the FDA nor its Cardiovascular and Renal Drugs Advisory Committee voiced concerns about the appropriateness of the presentation’s statistical philosophy. Additionally, although the CardioRenal AdCom’s first recommendation to the FDA was negative, the combination’s efficacy and its analysis was not the reason. I regarded Pravigard Pac to be a major positive chapter in the Bayesian story, notwithstanding the fact that the product was not a financial success.

We built Bayesian time-to-event models for the meta-analyses, where the primary event considered was any myocardial infarction [47]. We considered three multivariate models, with the most general one assuming non-proportional hazards. In particular, we did not want to stake everything on a model that assumed constant hazard over time. For example, one of the two agents might carry the combo’s efficacy early on, say in the first year, with the other agent taking over for the later term. If that were the case, then a combo would not be necessary. Patients could be given the first agent for the first year and then switch to the other agent, avoiding the side effects of the other agent. Our model 3 allowed for different hazards in different years.

**Figure 4:**
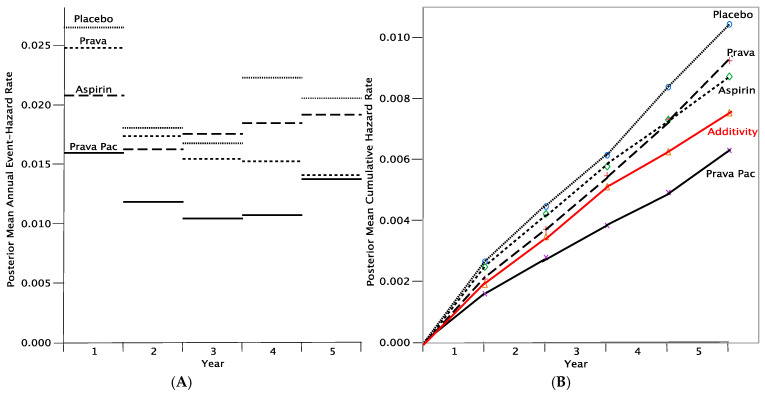
(**A**) Posterior means of the annual hazard rates by treatment. See Berry et al. [47] for the model description and its table 3 for the numerical values of the means and standard deviations. (**B**) Posterior means of the cumulative hazard rates for the four treatments [47]. The Additivity curve does not correspond to any treatment regimen. Rather, it represents the hypothetical results of a combination of the two single agents as though they were statistically independent. Namely, it is simply the sum of the means of the single-agent effects shown in (**B**). Numerically, it is the sum of the Prava and Aspirin curves minus the placebo curve.

Figure 4 shows that it is not possible to give one agent at a time and still receive the benefits of the simultaneous combination [47]. Both agents contribute to the effect of the combination throughout the 5-year period of follow-up. In fact, and somewhat surprisingly, comparing the Additivity and Pravigard Pac curves in Figure 4B shows that the sum of the effects of the single agents is markedly less than the efficacy of the combo. In other words, there is evidence for substantial synergy. Table 1 indicates that the posterior probability that Pravigard Pac is effective, that is, superior to placebo, over the 5-year period is 0.9992 [47]. Moreover, the probability the combination is synergistic in these 5 years is 0.9332, a calculation not possible in the traditional statistical approach.

This example demonstrates the importance of not relying on proportional hazards assumptions. Indeed, in Berry Consultants we generally use a non-proportional model in time-to-event trials, including GBM AGILE—see below.

7. **Eli Lilly’s phase 3 AWARD-5 trial for Trulicity** **^®^, type 2 diabetes**

The AWARD-5 trial (ClinicalTrials.gov NCT00734474) was the first phase 3 trial of dulaglutide (LY2189265, Trulicity^®^) for treating type 2 diabetes [48,49]. Eli Lilly and Berry Consultants (Indianapolis, IN, USA,) designed the trial in collaboration with the FDA as part of the Critical Path Initiative of CDER and CBER [48,49]. The trial was wholly Bayesian and adaptive. It accrued patients between 2008 and 2011. The trial antedated the FDA’s Complex Innovative Design (CID) initiative but it might be regarded to be the first complex innovative design [48,49]. AWARD-5 introduced some of the innovations that are now included in phase 3 platform trials such as GBM AGILE.

The AWARD-5 design called for a seamless shift (without pausing accrual) between its Stages 1 and 2. Stage 1 was adaptively randomized dose-finding among seven positive doses of dulaglutide versus two controls, placebo and sitagliptin [7,48,49]. The dulaglutide doses considered were 0.25, 0.50, 0.75, 1.00, 1.50, 2.00, and 3.00 mg. Stage 2 was fixed randomization of the two positive doses that were chosen from the seven positive doses investigated in Stage 1 compared against the two controls.

The trial had bi-weekly interim analyses during its Stage 1. The decision to shift to Stage 2 and its timing were based on Bayesian predictive probabilities assessed at these analysis epochs. In practice, the shift occurred at the earliest time allowed by the protocol and the design algorithm chose the smallest allowable sample size for Stage 2, again based on Bayesian predictive probabilities. The trial’s design algorithm called for choosing two non-adjacent doses. The doses selected (0.75 and 1.50 mg) became the two marketed doses of dulaglutide. The drug became the best-selling drug in diabetes, and it led the current international craze for GLP-1 agonists for weight loss as well as for diabetes, even though dulaglutide was never approved by the FDA for weight loss.

The first stage of AWARD-5 was similar to the ASTIN trial (see above), including using adaptive randomization and modeling dose-response using NDLM, but it had important additional features. The goal of the first stage was to determine whether dulaglutide was effective in type 2 diabetes and, if so, at what dose or doses. When the algorithm picked two doses—or potentially only one dose—the trial would shift seamlessly into its Stage 2. The selected dulaglutide doses were not allowed to be adjacent. Stage 2 would include those two doses and the controls with fixed randomization probabilities. The seamless shift alone saved at least a year of development time compared with running a phase 2 trial and then setting up to run a separate phase 3 trial.

We designed AWARD-5 to assess efficacy and safety measures based on prior clinical pharmacology studies. We did this by developing a clinical utility index (CUI) that served as the trial’s primary endpoint. The CUI had four components: HbA1c (the standard registration endpoint in diabetes), weight loss, and safety endpoints heart rate and diastolic blood pressure [7,48,49]. We built longitudinal models for each of these components. In the actual trial the longitudinal modeling was effective and efficient. It helped the design algorithm to select two doses, 0.75 mg and 1.50 mg, and to do so as soon as it was allowed. The algorithm moved into its Stage 2 when it picked the two doses and it selected the two doses when no patients had reached the defining 12-month visit [48,49].

Revisions of the adaptive randomization probabilities in the trial’s Stage 1 occurred biweekly and were monitored by a Data Safety Monitoring Board (DSMB). The randomization probabilities established by the algorithm favored assigning newly enrolling patients to the better-performing investigational dose regimens on the basis of the CUI. This provided the trial with more information about the better performing dose regimens while also providing better treatment to patients in the trial. Other doses had lower probabilities of being assigned, but some of the other doses had positive probabilities so as to not miss an effective dose that happened to be outside of the currently most likely range of best doses.

An incident in the AWARD-5 trial was revealing. During the actual trial, I served as its independent statistician reporting to the DSMB. I had made clear to the DSMB members that they could not take any actions that would deviate from the trial’s design algorithm—except regarding matters of safety. At one of our meetings they said, “We know we cannot overrule the decisions that the algorithm has made and is making. But if we were able to make the decisions instead of the algorithm then we would do exactly what the algorithm has done.” That reaction was testament to the work we had put into building the algorithm and the trial’s CUI.

We ensured control of type I error and statistical power using simulations across a range of null and alternative hypotheses. We simulated hundreds of different scenarios, including longitudinal models that generated results that were different from the models used in the design’s algorithm.

After the algorithm had selected the two doses, 0.75 mg and 1.50 mg, for the AWARD-5 Stage 2, Lilly initiated other phase 3 trials using those two doses of dulaglutide against other comparator diabetes drugs. In 2014 the FDA and the EMA approved Trulicity^®^ for type 2 diabetes at the two doses selected in AWARD-5. Trulicity^®^ quickly became Lilly’s best-selling drug, peaking at USD 7.1B revenue in 2023. This was despite a worldwide shortage of the drug in 2022 that continued into 2025. In 2020 the FDA extended the approval of Trulicity^®^ for diabetes to two higher doses (3.0 mg and 4.5 mg) based on the AWARD-11 trial [50,51,52]. The 3.0 mg dose in AWARD-5 showed a mean weight loss of 4.45 kg (10 lbs) [48,49]. This observation was predictive of and similar to the mean loss of 4.0 kg (8.8 lbs) at the same dose in AWARD-11 [50].

Trulicity^®^ is a GLP-1 agonist. It set the stage for the modern use of GLP-1 agonists for obesity as well as for diabetes, even though Trulicity^®^ has never been approved for obesity. GLP-1 agonists are being evaluated in other diseases. An example is non-alcoholic fatty liver disease [53].

8. **I-SPY 2, prototype adaptive platform trial in neoadjuvant breast cancer**

I-SPY 2 was a phase 2 adaptive Bayesian platform clinical trial in neoadjuvant breast cancer. It ran from 2010 to 2022. I-SPY 2 utilized many statistical innovations, as I will briefly describe here. The innovations were enabled by the Bayesian approach, and indeed that approach is itself new or unknown in most fields of medical research. I have described specifics about the trial’s design elsewhere [5,6,7]. I will focus on how it was possible to introduce radical innovations in the context of regulatory involvement and the inherent conservative nature of drug development, and given the similarly conservative tradition of clinical trial design in the 1900s to early 2000s.

Laura Esserman was the PI of I-SPY 2. I designed the trial and served as its co-PI. Dr. Esserman was an enthusiastic champion and fellow disrupter. Her enthusiasm was essential to the trial’s success. I-SPY 2 with its many statistical innovations would not have worked without her championship and enthusiasm. Two other critical people who were instrumental in the formative stages of I-SPY 2 were Janet Woodcock, Director of the FDA’s CDER, and Anna Barker, former Deputy Director of the National Cancer Institute.

I-SPY 2’s design has served as a prototype for other platform trials in a variety of diseases, including registration trials in oncology (see below). Descendants of I-SPY 2 utilized its innovations and added other innovations. The I-SPY 2 design and those of its descendants are complicated and were made possible by modern improvements in Bayesian statistical software and computer hardware. As indicated earlier, the U.S. FDA (CDER and CBER) recognizes complicated designs for registration under the rubric complex innovative designs (CIDs) [54,55]. I-SPY 2 was a prototype for CIDs, even though it was not itself a registration trial.

How a revolutionary trial such as I-SPY 2 came into being is arguably more important than what the trial was. In a sense it was itself an experiment. I ignored history and the Belmont Report. I started from scratch, thinking multiarmed bandit problems all the while. I was encouraged by Drs. Esserman, Woodcock, and Barker, and we had initial funding from the Foundation for the National Institutes of Health. The trial’s design was based on multiarmed bandit problems as described in Section 3 above. Treating patients with the better-performing therapies while learning efficiently and accurately regarding the effects of the various therapies was my motivation in the trial’s design. See Section 3, bandit problems, for my description of conflicting desiderata. Especially important was having effective treatment of patients in the trial as a primary goal [16,27]. The experience of using response-adaptive randomization in scores of trials at M.D. Anderson was essential [25], as was the support of the FDA for I-SPY 2 and beyond I-SPY 2, especially from Dr. Woodcock.

An obvious focus of Figure 3 is the role of the U.S. FDA. Innovations in drug development are not possible without support or at least some level of acceptability from the FDA. In the case of GBM AGILE, the FDA has encouraged and indeed has led innovations. I-SPY 2 has been promoted by the FDA. For example, in an article on master protocols, Woodcock and LaVange use I-SPY 2 as a prototypic example [55]. Further, they indicate that “Innovative aspects of the I-SPY 2 trial design include response-adaptive randomization to assign patients to the most promising treatment or combination of treatments in their respective molecular breast-cancer subgroups … while maintaining a sufficient number of patients assigned to the standard of care, shared use of control patients across treatment comparisons, and Bayesian decision rules to determine whether or when therapies with low probabilities of success or side effects should be discontinued and therapies with high probabilities of future success … should advance for further study.” [55].

However, I did not attempt to find an optimal bandit strategy in I-SPY 2. The main reason was that the optimal strategy would be incredibly complicated, especially when *k* > 2, when *k* varies with time, when the patient horizon *N* is unknown [13], when outcomes are delayed, and in the context of covariates [56]. Exact computations for finding an optimal treatment assignment strategy using dynamic programming (backward induction) and that account for these complications would have been impossible.

Most importantly, I wanted to include some element of randomization, which would make optimality calculations even more complicated [57]. Randomization was a necessary attraction for the medical research community, including regulators and the very important 18 pharmaceutical companies that provided funding for the trial and contributed the 23 investigational therapies that were evaluated in the trial.

The I-SPY 2 trial utilized many innovations that were motivated by and facilitated by the Bayesian statistical approach. The trial was successful in utilizing these innovations for the benefit of patients both in the trial and following the trial. Nine of the twenty-three investigational therapies graduated ready for phase 3, representing most of the trial’s 10 prospectively defined molecular marker-defined signatures. Four of the nine graduates have since received marketing approval within molecular subtypes of adjuvant or neoadjuvant breast cancer, but there is something for everyone (see Table 2). The investigational therapies that did not graduate had a thorough phase 2 evaluation in the various molecular subtypes of the disease, and with a large control group.

The co-primary focuses of I-SPY 2 were understanding the best use of candidate therapies in early breast cancer, doing this much faster than standard trials, and delivering better medicine to patients participating in the trial. The trial accomplished all three goals, including treating patients better by assigning fewer patients to ineffective therapies.

In 2022 I-SPY 2 ended and was replaced by a very different trial called I-SPY 2.2 that I did not support [58].

**Table 2:** Innovative features of I-SPY 2.

Adding and dropping arms. I-SPY 2 was designed to be potentially never-ending, although it did end in 2022. An arm in the trial stopped accruing patients when it graduated to phase 3, stopped accruing patients for futility, or reached a predetermined maximum sample size. In all cases, we followed patients and kept confidential the fact that the arm had stopped accruing patients until all the arm’s patients were through surgery.
2. Collection of basket trials. Patients were assigned to one of 8 subtypes defined by 3 molecular tumor markers: hormone receptor status (HR), HER2 receptor status (HER2), and MammaPrint (MP). Investigational arms were evaluated in up to 10 signatures that are combinations patient subtypes and that are possible clinical indications.
3. Definition of type I error. The basket aspect of each investigational arm of I-SPY 2 means that there are several possible types of error for each arm. For example, an arm may graduate in a signature for which some subtypes are correctly positive but others are incorrectly positive. That is not a type I error. In I-SPY 2, I defined a false-positive conclusion to be only when the concluding signature contains no patients who will benefit from the therapy.
4. Bayesian predictive probability of success in a future phase 3 trial. Adaptive randomization was based on predictive probabilities. Stopping accrual because of graduation to phase 3 or futility was based on the Bayesian predictive probability of future success. As a numerical example of the benefits of adaptive randomization, including its role in treating patients in the trial more effectively, consider neratinib. It graduated in the trial’s HR–/HER2+ signature with a Bayesian-modelled pCR rate 56% versus the control’s 33%. In the bigger signature “any HER2+” the respective comparison was 39% versus 23% [59]. The rate of pCR over all 115 patients that were assigned to neratinib across the 8 subtypes was 37%. These subtypes have different prevalences and different sensitivities to chemotherapy. For patients assigned to control the pCR rate was 20%. Two aspects contribute to the 17% difference. One is the higher pCR rate of neratinib averaged over the 8 subtypes. The other is the trial’s adaptive randomization, with greater probability of assigning neratinib in subtypes more likely to benefit from the drug. Had neratinib been assigned equally across the disease, just as the case for control, the estimated pCR rate on neratinib would have been 27%. As such, 7% of the 17% was due to neratinib and 10% was due to adaptive randomization. In the circumstances of the actual trial, the benefit due to adaptive randomization was greater than 15%. The reason for the added 5%+ is that, for subtypes in which neratinib was performing poorly, the alternative assigned by adaptive randomization was not control therapy. Instead, 80% of the patients would have received other experimental arms, including veliparib + carboplatin (VC). The VC arm graduated in the triple-negative breast cancer signature with a 51% pCR rate versus 26% for control [60] (if 15% conversion of non-responders to a pCR were due to a drug, say, then that would be clinically important and worthy of further development). In a phase 3 trial called ExteNET (NCT00878709), neratinib versus placebo for 12 months (extended therapy) was shown to prolong disease-free survival in lymph-node-positive HER2+ breast cancer following trastuzumab-based adjuvant chemotherapy [61]. This indication received FDA marketing approval [62].
5. Continuous learning and Bayesian updating. Each month we calculated the current distributions of pCR rates for all subtypes and all possible signatures for all arms in the trial. We used these distributions to calculate predictive probabilities and, in turn, the assessment of each investigational arm’s status in the trial. This included monthly updates of each arm’s randomization probabilities and decisions regarding graduation to phase 3, dropping for futility, and stopping accrual at the arm’s maximum sample size.
6. Common control arm (by patient subtype). Investigational arms in I-SPY 2 were compared against a common set of controls, depending on patient subtype. The final analysis of each investigational arm was its Bayesian probability of superiority to control for the primary endpoint of pCR for each signature.
7. Time machine [63,64]. Patients were assigned to the control arm with 20% probability unless there was one investigational arm in the subtype, in which case randomization was 1:1. The control cohort for an investigational arm includes all concurrently randomized controls plus all previously randomized controls. However, the outcomes of the non-concurrently randomized controls were adjusted and partially discounted for time trends in the trial. The time trends were assessed for all the arms in the trial, including the other investigational arms. The result was a databank of controls that was typically an order of magnitude greater than the number of patients in the investigational arms, with statistical power much, much greater than typical phase 2 cancer trials.
8. Longitudinal model of disease burden as auxiliary end point [5]. We built a model that related reductions in tumor volume during neoadjuvant treatment, depending on patient subtype. We used historical data from I-SPY 1 but updated the model using I-SPY 2 patients who had experienced surgery. At each analysis epoch we calculated the probability of achieving pCR for each patient in the trial who had not yet had surgery. We used multiple imputation to find the probability distributions of all the pCR rates for the arms in the trial [6,7].
9. Nested partial factorials. The continuing control arms of I-SPY 2 enabled addressing combination therapies. One of the initial arms in the trial was AbbVie-sponsored veliparib + carboplatin (VC) [60]. Its successful graduation led to a phase 3 trial that isolated the effects of V and C [65].
10. Use of computer simulation to assess design operating characteristics. Traditional statistical designs of clinical trials include type I error rate and statistical power. They should also be addressed in Bayesian trials, especially type I error, if only to preserve continuity with traditional trials.

9. **FDA/NIH innovative-trials grant to the NETT and Berry Consultants**

A principal focus of Figure 3 is the leadership role played by the FDA in clinical trial innovation. The figure cites relevant guidances for industry that the FDA has issued. It also refers to a Regulatory Science Program co-funded by the FDA and NIH. I was a co-PI of the grant. The other co-PIs were William Barsan of the University of Michigan and the Neurological Emergency Treatment Trials Network (NETT), an NIH-sponsored cooperative group, and Roger Lewis of UCLA and Berry Consultants. One of the projects funded by the grant was titled “Adaptive Designs Accelerating Promising Trials Into Treatments (ADAPT-IT).” The project ran from 2010 to 2015. The advisory board for the grant consisted of FDA medical scientists and the lead statisticians from the FDA’s CDER, CBER, and CDRH [66,67]. As part of ADAPT-IT, Berry Consultants designed five Bayesian adaptive trials that would be conducted by the NETT [66,67].

An important part of the grant was addressing the barriers associated with introducing Bayesian and adaptive approaches into clinical trials. These are some results, as stated in the Abstract:

“[R]espondents perceived clinicians to be less likely to understand ACTs [Adaptive Clinical Trials] and that ACTs probably would increase the efficiency of discovery. Textual and focus group responses emerged into several themes that enhanced understanding of [visual analog scales] attitudinal data including the following: acceptability of adaptive designs depends on constituency and situation; there is variable understanding of ACTs (limited among clinicians, perceived to be higher at FDA); views about the potential for efficiency depend on the situation and implementation. Participants also frequently mentioned a need for greater education within the academic community. Finally, the empiric, non-quantitative selection of treatments for phase III trials based on limited phase II trials was highlighted as an opportunity for improvement and a potential explanation for the high number of neutral confirmatory trials.”

[67]

The AWARD-5 trial and I-SPY 2 described above ran concurrently with this grant. They both took advantage of the “opportunity for improvement” in the failure rate of phase 3 trials. In particular, AWARD-5 seamlessly combined phases 2 and 3, and I-SPY 2’s focus was on success in a phase 3 trial. Moreover, these two trials led to the seamless-phase GBM AGILE trial in which patients assigned to investigational agents in their adaptively randomized stage count fully for drug registration.

10. **Eisai phase 2 Leqembi^®^ 201 trial perfectly predicts phase 3**

An article titled “Improving Alzheimer’s disease phase II clinical trials” says “the Alzheimer’s Association convened a Research Roundtable on 23 and 24 June 2011, in Washington, DC, bringing together scientists from academia, industry, and government regulatory agencies to discuss strategies for improving the probability of phase II trial results predicting success when considering the go/no-go decision-making process leading to the initiation of phase III.” [68]. I was asked by the organizers of the referenced roundtable to present a Bayesian adaptive approach addressing the subject of the conference. Veronika Logovinsky of Eisai Co., Ltd. (Tokyo, Japan) was in attendance. After the conference she asked me to help Eisai design a phase 2 trial for BAN2401, also known as lecanemab and Leqembi^®^, that would avoid the mistakes being reported and criticized at the conference. Lecanemab is a monoclonal antibody used to treat Alzheimer’s disease. Working through Berry Consultants, Scott Berry and I complied with her request [69,70].

Lecanemab trial 201 considered six dose regimens, including placebo. At the trial’s outset, 2/7ths of the patients, approximately 29%, were randomized to placebo. The remaining 5/7ths were randomized equally, so 1/7th of the patients were assigned to each of the five positive doses of lecanemab. A traditional approach would have continued with these randomization probabilities throughout the trial. Our design included 14 interim analyses, approximately equally spaced, with the first occurring when the 196th patient accrued. The principal goal of these analyses was to learn as much as possible about the efficacy of the dose compared with placebo that would eventually be used if there was a phase 3 trial—subject to a total maximum sample size of 856 patients. The proportion assigned to placebo was reassessed at each interim analysis and was the same as that for the currently most likely phase 3 dose [69,70].

Early in the trial, the two highest doses performed well, leading to greater sample sizes for these doses. Their final sample sizes were 253 and 161, which was 48% of the total. Only 51 and 52 patients (a total of 12%) were assigned to the two lowest doses. Placebo was assigned to 247 patients, at 29% of the total. This emphasis on the highest doses and de-emphasis on the lowest doses meant the trial was more informative about the dose regimens of greatest interest.

The trial’s design easily handled the greater than 30% rate of missingness typical of Alzheimer trials. A traditional approach for imputing missing data is “last observation carried forward” or LOCF. In the actual trial this approach would have concluded a 13% reduction in disease progression in comparison with placebo [70]. Eisai would not likely have run a phase 3 trial with such a weak signal, and an effective drug would have been shelved. Using multiple imputation in the prospective Bayesian approach led to an estimated 27% reduction in disease progression. This is clinically meaningful, and it was sufficient to persuade Eisai to “go to” phase 3. It was also sufficient for the FDA to grant accelerated approval [71]. Referring back to the goal of the 201 trial, predicting whether a phase 3 trial would be a good gamble, and at the best dose, the phase 3 trial showed the same 27% reduction at the highest dose, and led to full approval from the FDA [72]. Of course, the fact that the prediction was perfect was somewhat accidental; both were point estimates, with the phase 2 trial having a Bayesian probability distribution and the phase 3 trial having a confidence interval.

The prospectively defined Bayesian model did much better than the old-fashioned LOCF because it was trained to consider each patient’s patterns of data, and data missingness in particular. The algorithm learned what patterns of disease led to dropping out. It learned that dropouts of placebo patients were largely due to the treatment not working.

The patterns of missingness in patients assigned to the highest dose were mostly due to the mandate by an outside force unrelated to patient outcomes. Namely, partway through the trial an ex-U.S. regulatory authority required that the highest dose no longer be used for an important subset of patients in the trial: those who were APOE4 carriers. Further, this authority mandated that protocol therapy on the highest dose stop for APOE4 carriers who had been receiving that dose for less than 6 months. This circumstance led to very high rates of missing data at this dose, despite the fact that the patients were doing relatively well [70]. Although these patients’ last observations tended to be favorable relative to patients with the same time on therapy on other doses, in comparison with the Bayesian model, LOCF did not adequately account for their future results.

There is an additional advantage of running an efficient, informative, adaptive phase 2 trial. Namely, a good estimate of efficacy from a well-designed phase 2 trial serves to right-size the phase 3 trial. Eisai had a phase 2 estimate of efficacy that turned out to be perfectly predictive in phase 3. As such, their phase 3 trial was neither too big, thus saving time and resources, nor too small, thereby adequately powering the phase 3 trial and showing statistical significance which was the basis for the drug’s marketing approval. Trial 201 enabled building a Goldilocks phase 3 trial [72].

11. **IMI-EPAD (Innovative Medicines Initiative, European Prevention of Alzheimer’s Dementia)**

The I-SPY 2 trial set a standard for Bayesian platform trials. For example, this is from the Innovative Medicines Initiative (IMI) in 2015:

“Inspired by the I-SPY initiative, the EPAD consortium will develop an adaptive design in a proof-of-concept trial for early intervention in Alzheimer’s disease. Ten pharmaceutical companies are investing in this ambitious project, which will address many challenges regarding the selection of patient sub-groups, drug candidates, optimal end points and statistical methodology. Regulators will be consulted at an early stage to ensure their buy-in for this unprecedented endeavour in the field of dementia.”

[73]

Despite the optimism, the EPAD story is mainly negative, which is why I did not include it in Figure 3. However, it made important contributions and helped us understand the difficulties in setting up a platform trial. The IMI (now IHI, for Innovative Health Initiative) arranged for generous funding for the EPAD consortium that included Berry Consultants and that the IMI selected in a competitive process. From 2015 to 2020 the EU contributed EUR 26 M and the European Federation of Pharmaceutical Industries Associations (EFPIA) contributed another EUR 27 M of the total EPAD funding of EUR 59 M [74]. Nevertheless, the development of EPAD foundered. It failed in October 2020 [74].

According to their website, EPAD was a collaborative research effort of 39 partners across Europe. There were identifiable reasons for EPAD’s ultimate failure, but I do not know the details. However, the impact of the COVID-19 pandemic could not have been positive, nor was Brexit. Further, although having 39 partners in a trial may be wonderful from several perspectives, a developmental process can be dysfunctional if some of the partners are competitors and have unequal shares in making the project work. Simply put, there can be too many cooks.

Platform trials have many stakeholders. A lesson from the EPAD story is that it is difficult to build a platform trial that promises rewards for every stakeholder. Adequate funding from philanthropy, government, and industry is essential but not sufficient. A corollary is that the design must be flexible in terms of patient population and patient subpopulations for the individual treatment arms, and the design should be flexible in terms of statistical issues, such as sample size and statistical power. Along these lines, the trial could be registrational for some arms, pre-registrational for other arms, and post-registrational for still other arms. Although the trial must have a master protocol, each treatment arm that enters the trial must have its own appendix to the master protocol. The master protocol should state explicitly which aspects of an arm’s appendix can supersede those aspects of the master protocol.

The final chapter may not have been written in the EPAD story:

“EPAD was unable to initiate a proof of concept trial, but they developed a trial-ready proof-of-concept platform that was capable of running phase 2 clinical trials with research participants with preclinical and prodromal Alzheimer’s disease, with biomarker evidence of Alzheimer’s disease pathology. The platform was open for expressions of interest from pharmaceutical and biotechnology organisations, academic researchers, and funders who had suitable interventions ready for testing.”

[74]

“The platform was designed to efficiently deliver early, accurate results. EPAD studied new drugs in a well-designed phase 2 PoC trial with clinical endpoints, using the power of adaptive design and Bayesian statistics. The EPAD trial platform infrastructure is fully operational.”

[74]

12. **ROAR and other basket trials**

Woodcock and LaVange describe three types of master protocols: “Master protocols may involve one or more interventions in multiple diseases or a single disease, as defined by current disease classification, with multiple interventions, each targeting a particular biomarker-defined population or disease subtype. Included under this broad definition of a master protocol are three distinct entities: umbrella, basket, and platform trials.” [55]. A basket trial is “a single targeted therapy in the context of multiple diseases or disease subtypes.” [55,75]. The goals and setting of basket trials are perfect for a Bayesian approach. Unfortunately, this was not appreciated by the ROAR investigators or by the eventual sponsor of the trial (see below). It was a great opportunity that was missed. Fortunately for the sponsor and patients, the FDA made up for the missed opportunity even though the FDA’s analyses were not formally Bayes.

Consider a therapy to be evaluated in two tumor types. Suppose type A is evaluated first and the therapy is either effective or not in type A. What about tumor type B? Is the therapy effective in B? Of course, we do not know, but whatever was our probability of efficacy for B, it increases when the therapy is effective in A and it decreases when the therapy is not effective in A. The average of the two probabilities (weighted by their predictive probabilities) is the prior probability of “efficacy” for tumor type B.

Now suppose the therapy has been evaluated in ten different tumor types and is found to be effective in all 10. What about the 11th tumor type? It seems likely to be effective in the 11th tumor type, but it is not a sure thing. For example, tumor type 11 might have different sensitivity than the others.

Bayes’ theorem can be used to predict the result in the 11th tumor type, and in one or both of the 11th and 12th tumor types, etc. This is true even if no patients with tumor types 11 or 12 were treated with the therapy. Apropos of pan-tumor approvals in basket trials, the probability of effectiveness in all 12 tumor types can be calculated for any interim results, and the same is true for any cluster of the 12 tumor types.

Using Bayes’ theorem requires a hierarchical model where “tumor type” is one of the levels of experimental unit. “Patient” is another level, one nested within tumor type. A hierarchical model is natural for this setting. Moreover, mathematically speaking, it is essential to use such a hierarchical model, one requiring borrowing results across tumor types. This was demonstrated by Charles Stein in the 1950s [6,7,76,77,78]. He showed that failing to borrow across tumor types was “inadmissible.” This is a very powerful result. It means that there are uniformly better estimates of the true response rates regardless of the true values. In particular, Bayesian estimates that borrow results from neighboring tumor types are uniformly better than no borrowing. The borrowing should be stronger for settings that are a priori homogeneous, such as different tumor types that harbor the same genomic biomarker, but as James and Stein showed, borrowing in the context of heterogenous populations is better than not borrowing at all [78].

**Figure 5:**
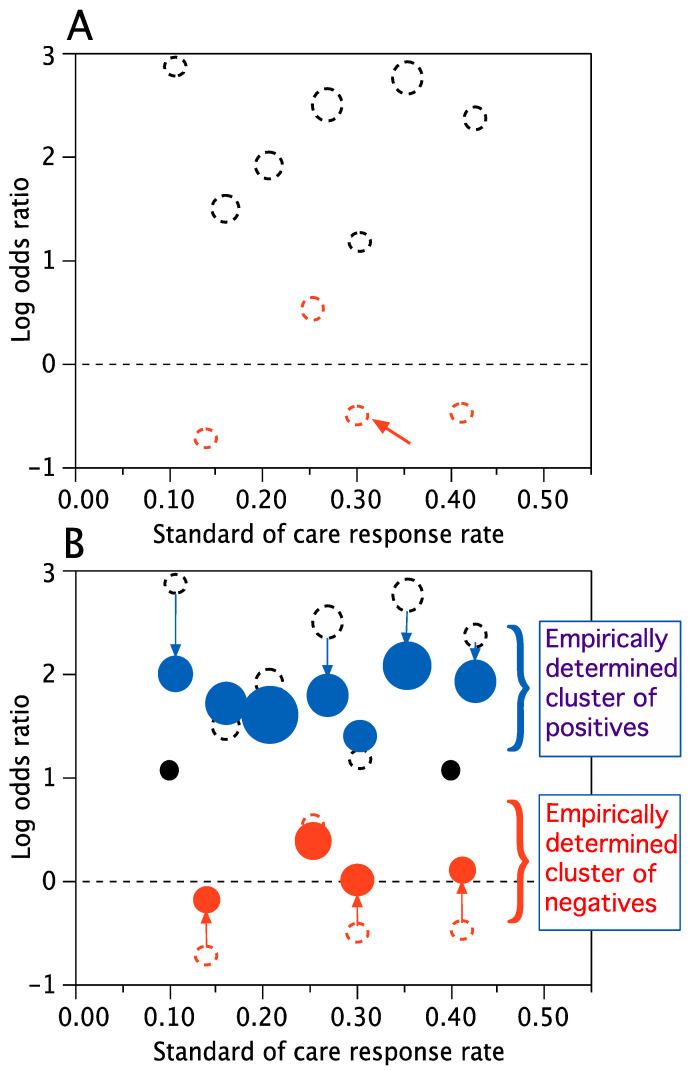
(**A**) (These data are not real and are neither from ROAR nor any other trial.) Hypothetical example, representing shrinkage of estimates to each other, especially those within the same cluster, and representing expansion of the “sample size” of each tumor response rate estimate. Each dot represents a tumor type. The horizontal dimension is the tumor type’s comparison SOC response rate based on historical information. The vertical dimension is the log odds ratio of investigational therapy versus SOC. The areas of the circles are proportional to sample size. The dashed horizontal line has a log odds ratio equal to 0. A dot on this line would mean the investigational therapy’s response rate in the respective tumor type is the same as that for SOC. The dashed open circles are the raw estimates. For example, the arrow points to a tumor type with SOC response rate of 0.30, the raw data might have been 6 responses out of 29 patients, or 20.7%. The odds ratio for this tumor type is (6/23)/(0.3/0.7) = 0.609 and the (natural) log of 0.609 is equal to −0.50. (**B**) The solid circles are the Bayesian model estimates based on borrowing from the efficacies of the investigational arm across all tumor types, with the amount of borrowing determined by the data and the hierarchical model. In the tumor type indicated by the arrow in plot A, the solid circle is on the horizontal dashed line at 0 and so the model estimate increased the log odds ratio estimate for this tumor type from −0.50 to 0, and the response rate estimate from 20.7% to 30%. In addition, for the ROAR trial we used formal modeling of clusters of positive and negative tumor types, with borrowing within clusters as well as across clusters. As such, the ROAR model had a three-level hierarchy. The arrows and the consequent solid circles show how the raw estimates are adjusted by the model. There are two kinds of adjustments. One is that the dots move along the arrows, sometimes called shrinkage. The other is that the dots typically increase in size. The dashed circles show actual sample sizes. For the solid circles the areas are “equivalent sample sizes” based on hierarchical borrowing from increases in response for neighboring tumor types, especially those in the same cluster, so larger circles imply smaller probability intervals. In the figure, the areas of the solid circles have more than doubled in comparison with the dashed circles, reflecting the greater precision due to borrowing. Raw estimates for tumor types with small sample sizes, further from the overall mean, and further from the cluster mean are regressed further. Tumor types with raw estimates further from the cluster mean (in the vertical direction) borrow less and therefore have less increase in precision (area of the solid circle). Two tumor types that have solid black circles have 0 sample size. One has a historical response rate of 0.10 and the other 0.40. They have no dashed circles associated with these tumor types because they have no data in the trial. Their solid black circles are small because their equivalent sample sizes are small—but they are not zero. Their position in the vertical direction is at the overall mean of the 11 tumor types that do have data regarding treatment effect. Like all the dots in the figure, the locations of the black dots are point estimates in the vertical direction.

Each tumor type has its own response rate (or log odds ratio of investigational versus control response rates), one that is selected from the population of response rates: *ρ*_1_, *ρ*_2_, …, *ρ*_10_, *ρ*_11_. This seems like a traditional statistics problem of sampling from a population, but it is not traditional. The reason is that the values of the *ρ*’s are not observable, but information about all the *ρ*’s is available via the observed responses. In the Bayesian approach, a hierarchical model requires a probability distribution of the *ρ*’s. Therefore, a Bayesian hierarchical model is a probability distribution of probability distributions [5,6,7,76,77,78,79]. Information accrues about the distribution of *ρ*’s as well as about the individual *ρ*’s themselves, hence the advantage of borrowing across the results of the various tumor types (see Figure 5).

Berry Consultants has designed many Bayesian adaptive basket trials in cancer using hierarchical models. One of the most interesting was ROAR, a trial sponsored by and originally designed for GlaxoSmithKline (GSK, London, UK). The investigational therapy was the combination of two of GSK’s drugs, dabrafenib and trametinib, which are both tyrosine kinase inhibitors that target specific kinases within the mitogen-activated protein kinase (*MAPK*) pathway. Eligibility for ROAR was restricted to patients having rare tumor types and cancers that have *V600E* mutations of the *BRAF* gene. I met several times with GSK and the FDA regarding the trial’s Bayesian design. The FDA was helpful, and naturally enough, they were especially interested in the design’s borrowing aspect.

In 2014–2015, as ROAR was accruing patients, GSK sold much of its cancer pipeline to Novartis (Novartis Pharma AG, Basel, Switzerland). The sale included dabrafenib and trametinib. Although I had worked with Novartis in designing an innovative series of Bayesian basket trials called the Signature program [79], their ROAR team lacked appreciation for Bayesian approaches and had essentially no understanding of ROAR’s Bayesian design and its full potential.

I met with Novartis and the FDA regarding the eventual approval of the combination for one of the tumor types considered in ROAR, namely, anaplastic thyroid carcinoma (ATC). In discussing how to report the ROAR trial results, the FDA indicated that they would and the company should follow the protocol and use the estimates from the Bayesian hierarchical modeling of the primary endpoint, which was overall response rate (ORR).

Despite this very helpful (and appropriate) stipulation, Novartis and the trial’s investigators analyzed the trial’s results as though the design had been frequentist with each tumor type treated as a distinct trial. This is the very analysis that Charles Stein proved to be “inadmissible.” Not only were they ignoring the trial’s protocol and the FDA’s stipulation, they were providing estimates that were uniformly less accurate, no matter the underlying truth. What a shame!

Moreover, the protocol modeling they ignored would have provided the answer to what at the time was a natural question. Namely (based on ROAR and possibly other trials), what is the probability that the combination therapy is effective (increases rate of ORR) for all solid tumors having *BRAF V600E* mutations? See Figure 5. Not only was the Novartis investigators’ analysis inadmissible, it could not be used in addressing what turned out to be the right question.

Evidently, the trial results persuaded the FDA what was the right question, because they both asked and answered it: On 22 June 2022, the Food and Drug Administration granted accelerated approval to dabrafenib (Tafinlar^®^, Novartis) in combination with trametinib (Mekinist^®^, Novartis) for the treatment of adult and pediatric patients ≥ 6 years of age with unresectable or metastatic solid tumors with *BRAF V600E* mutations who have progressed following prior treatment and have no satisfactory alternative treatment options. Dabrafenib in combination with trametinib is not indicated for patients with colorectal cancer because of known intrinsic resistance to *BRAF* inhibition. Dabrafenib is not indicated for patients with wild-type *BRAF* solid tumors [80].

Whatever was the FDA’s reasoning, it had a strong component of borrowing. If their analysis had been Bayesian then they would have announced it. Moreover, their conclusion could not be supported by any traditional statistical calculations, so it was probably subjective (which is arguably Bayesian!). The probability that the combination is effective in the entire population of solid tumors that carry a *BRAF* V600E-mutation would be a straightforward calculation using a Bayesian hierarchical model. In the analog of Figure 5B, there would be a single cluster with all point estimates of the log-odds ratios greater than 0, including for tumor types not represented in the trial but represented in the figure by the solid black dots.

The trial’s investigators and Novartis published several articles of the results of ROAR [81,82,83]. All published estimates have been inadmissible. The first of the three references mentioned the Bayesian hierarchical approach, and it credited Berry Consultants [80]. However, the latter two articles make no mention of Bayesian methods, nor of hierarchical analyses, nor of borrowing. Rather, they published the results as though the trial’s protocol had specified nine independent trials having separate designs, with confidence intervals for the ORRs, and the confidence intervals are also inadmissible [82,83]. The protocol was Bayesian hierarchical borrowing and it was a pretense for the authors to assume otherwise.

13. **GBM AGILE (Glioblastoma Adaptive Global Innovative Learning Environment), a seamless adaptive Bayesian platform registration trial**

GBM AGILE was originally designed to be a phase 2 platform trial in GBM mimicking I-SPY 2 and its innovations in Table 2, with the principal exception that the primary end point is overall survival [84,85,86]. The trial’s genesis was a series of meetings between 2015 and 2019 hosted by Anna Barker, then of the National Biomarker Development Alliance and Complex Adaptive Systems at Arizona State University. Invitees were expert GBM researchers from across the globe, with concentrations in the United States, Canada, Europe, Australia, and China. During this series of meetings, a consensus developed that we should design and run an I-SPY 2-like trial in GBM.

In lieu of the eight molecular biomarker subtypes of neoadjuvant breast cancer in I-SPY 2, we included only three subtypes in GBM AGILE. These were newly diagnosed GBM with methylated MGMT (NDM), newly diagnosed GBM with unmethylated MGMT (NDU), and recurrent disease (RD). I-SPY 2 had 10 signatures. These are combinations of subtypes that are evaluated as possible investigational arm indications. The analog in GBM AGILE is five signatures, including each of the three subtypes plus ND (=NDU + NDM) and All (=NDU + NDM + RD). Further, pharma companies are invited to submit targeted drugs for consideration by GBM AGILE within enrichment biomarker subpopulations. This would mean additional subtypes and additional signatures (thus far GBM AGILE has not had an investigational therapy that included an enrichment biomarker).

The FDA felt that GBM was sufficiently different from breast cancer to mimic I-SPY 2. In particular, they worried about graduating an arm to a separate phase 3 trial should the arm perform very well. Perhaps running such a trial would be impossible. I proposed incorporating a small confirmatory extension cohort of 50 patients having the arm’s graduating signature that would be assigned to a Stage 2 within GBM AGILE. They agreed. Randomization to an arm in its Stage 2 would be fixed, typically at 40%. Stage 1 would be analogous to the I-SPY 2 trial, including adaptive randomization. The final analysis for arms that graduate to a Stage 2 would include the patients in its Stage 2 and also those in its Stage 1 in the arm’s graduating signature. Graduations would be seamless and would not be announced until the arm’s final analysis 12 months after the last patient was randomized to the arm. The totality of the results in such an arm’s Stages 1 and 2 would serve as a phase 3 registration trial.

**Figure 6:**
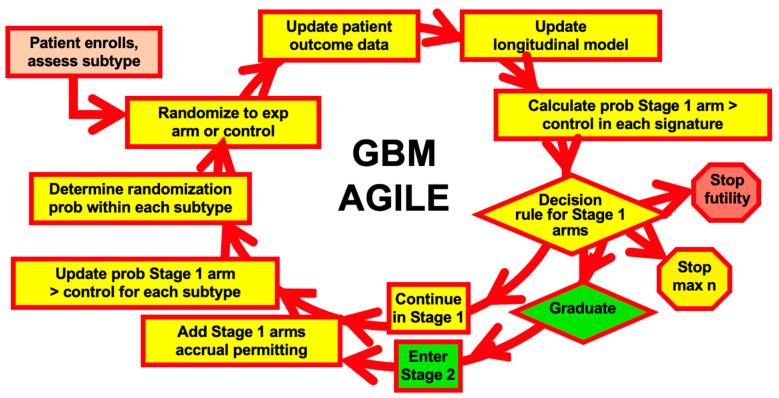
Never-ending circle of GBM AGILE. Stages 1 and 2 for each investigational treatment arm are the large concentric circles, reflecting the fact that an arm’s stage is not known by any of the investigators or clinical trial personnel without a need to know. Arms stop accruing patients for futility at any time after the 50th patient (over all subtypes) is assigned to the arm. Arms graduate to Stage 2 at any time after the 100th patient (overall) is assigned to the arm. The basis for futility and graduation is the arm’s predictive probability of success in the trial. Maximum sample size in Stage 1 (over all subtypes) varies by arm and is specified in each arm’s appendix.

Figure 6 shows the GBM AGILE design circle, indicating its continually dynamic nature and the merger of an arm’s Stages 1 and 2, for arms that have a Stage 2.

The results of the first four investigational arms in GBM AGILE have been announced and thus far none has graduated to a Stage 2. This is hardly surprising; failures in GBM clinical trials is the norm.

I have mentioned the FDA’s bold Complex Innovative Design initiative. It satisfied “in part, a mandate under section 3021 of the 21st Century Cures Act.” [54]. The CID initiative’s focus is innovation in phase 3 trials, which are pivotal trials used for marketing approval: “For the purposes of this guidance, CID includes trial designs that have rarely or never been used to date to provide substantial evidence of effectiveness in new drug applications or biologics license applications.” [87,88,89]. GBM AGILE served informally as a prototype CID even though it was not formally designated as a CID by the FDA.

The sponsor of GBM AGILE is the Global Coalition for Adaptive Research (GCAR), a non-profit company that was set up specifically to run that trial. GCAR has been so successful under CEO Meredith Buxton that it has become the go-to organization for running Bayesian adaptive platform trials.

14. **Precision Promise: Bayesian platform trial sponsored by the Pancreatic Cancer Action Network (PanCAN)**

Precision Promise was a sister trial of GBM AGILE in the sense of having a very similar design with the same primary endpoint, overall survival (OS), but in metastatic pancreatic cancer (PDAC). It was sponsored and run by the Pancreatic Cancer Action Network (PanCAN) [90]. It too worked with the FDA so that drug registration could be its goal. The patient population had two possible subtypes, first-line therapy and second-line therapy, and three possible signatures: (i) first line therapy, (ii) second line therapy, and (iii) both first and second line. Precision Promise had two innovations in addition to those of GBM AGILE.

A unique innovation in Precision Promise involved its control arm. PDAC had two standards of care when the trial started to accrue patients in 2020: gemcitabine plus nab-paclitaxel (GA) and modified FOLFIRINOX (mFF). Patients in clinical practice typically received GA or mFF in line 1 and then the other therapy in line 2 upon progression in line 1. The two regimens were regarded as having similar OS, although they had never been compared in an RCT. An investigational agent could enter the trial in combination with GA or mFF or with no backbone. The control arm we proposed was physician’s choice between GA and mFF. Quite reasonably, the FDA indicated that physician choice would not work because an investigational arm would have to be compared with its backbone, GA or mFF or both.

The proportion of patients assigned to investigational arms was 70%, with those available arms competing for patients via adaptive randomization. The remaining 30% was split 15:15 to GA:mFF, except that a patient could not be assigned to the same backbone or its control in both line 1 and line 2. If one control arm was counterindicated then the other control arm would be assigned with the full control probability of 30% (these rules were predictably difficult to manage in the trial).

I suggested to the FDA a type of compromise, a Bayesian-like compromise. We could use Bayesian hierarchical modeling by viewing GA and mFF as two members of a hypothetical family of standard of care arms. Consider an investigational arm with backbone GA. Hierarchical modeling would lead to mFF increase the precision associated with GA in comparing with the investigational arm. The net effect of borrowing from mFF is that if GA and mFF have similar OS in the trial then the effective proportion of patients assigned to GA is closer to 23% than the actual 15%.

The other unique innovation in Precision Promise is that line 1 patients who have progressed or are otherwise appropriate for moving to line 2 can be considered for rerandomized to a different arm of Precision Promise. We build a new methodology for sharing such a patient between the two arms that the patient received, one in line 1 and the other in line 2. I think the ability to rerandomize patients when the end point is overall survival has never been done before. The FDA agreed with our approach and our analyses. However, rerandomization was infrequent and it may not have been worth the complications caused in trial.

Unfortunately, Precision Promise attracted only two investigational arms between 2020 and 2024, Tyme’s drug SM-88 [91] and Fibrogen’s drug pamrevlumab [92]. Neither of these two investigational arms demonstrated a benefit. PanCAN decided in 2024 to contract with GCAR (the sponsor of GBM AGILE and the U.S. portion of REMAP-CAP/COVID and other platform trials) to continue running the trial. GCAR is the new sponsor (same as for GBM AGILE) and has decided to restart the trial using a new name: IMPACT PDAC [93].

15. **REMAP-CAP/COVID (*****Randomized, Embedded, Multi-factorial, Adaptive Platform Trial for Community-Acquired Pneumonia) with*** **COVID appendix**

The success of I-SPY 2 has led to many adaptive Bayesian platform trials. One of the most successful trials cited in Figure 3 was (and is) REMAP-CAP/COVID [94,95]. Working with global infection-focused research communities, Scott Berry of Berry Consultants led the design and analysis of REMAP-CAP/COVID. The initial development was in 2014, long before the COVID-19 pandemic, and was driven by the European Union, Australasia, and Canada. The United States joined in 2020 [94,95].

REMAP-CAP was registered in April 2016 (NCT02735707). Scott Berry had also led the design of a precursor design of REMAP-CAP. This was a US-funded trial for treating Ebola in East Africa [96], ClinicalTrials.gov Identifier NCT02380625. “The [Ebola] study was approved by US and Sierra Leone ethics committees, and reviewed by the US Food and Drug Administration. Additionally, data management, drug supply lines, and local sites were set up to handle an extensive epidemic. However, in response to the declining epidemic seen in February 2015, the trial was not initiated.” [96].

REMAP-CAP accrued about 500 CAP patients between April 2018 and March 2020 [97]. A primary rationale for and impetus for REMAP-CAP was to prepare the world for the next pandemic. On 9 March 2020, two days before the WHO declared COVID-19 a pandemic, REMAP-CAP exercised the “Adaptation during a Pandemic” section of its protocol by accruing its first COVID patient. Since that time, the trial has (adaptively) randomized more than 24,000 patients, more than 18,000 of whom were suspected or proven to have had COVID-19 [97]. The trial is ongoing. Investigators in 15 countries have completed or are currently evaluating 66 potential COVID interventions in 18 therapeutical domains [97]. REMAP-CAP investigators have had many high-impact, practice-changing publications, all of which are Bayesian [97].

## 6. Conclusions

The Bayesian approach has always had theoretical appeal. Over the last 20 to 30 years, and sporadically at first, the theory has been applied in designing actual clinical trials. Bayesian trials still represent a small minority, but their existence is changing the way investigators, regulators, and government and industry sponsors view innovation in clinical trials. A decision-analytic Bayesian approach aimed at delivering good medicine to a population is called a bandit problem. Bandit problems regard effectively treating patients who participate in trials as just as important as effectively treating patients in clinical practice who benefit from the trial’s outcomes. Such possibilities argue for the merger of clinical research and clinical practice, which means abandoning the Belmont Report.

By far the most important Bayesian contribution to clinical trials is the ability to observe the accumulating results and modify the future course of the trial on their bases. My tack in this article is that the “observer” is a computer armed with a prospective algorithm that dictates adaptations that were determined in advance of the trial. Any such design can then be simulated to calculate its type I error rate and statistical power.

The Bayesian clinical trials in Section 4 that have been designed since 1995 and that are still being conducted illustrate the use of and benefits of the Bayesian approach. The aspects of the Bayesian approach used in these trials are listed in Table 3. The “Bayesian characteristics” in the table are mostly self-explanatory. “Efficient” trials are those with a smaller sample size or trial duration in comparison with traditional trials. Trials 1, 4, 5–7, 9, and 12–15 indicate trials for product registration or for comparative effectiveness, that is, influencing treatments used in clinical practice.

I hope this article is helpful in guiding future clinical research. Integrating clinical practice with clinical research is one direction. Integrating real-world evidence with clinical trials is another. Integrating Bayesian methodology with machine learning is another. An area begging to be mined is precision medicine in clinical trials. I-SPY 2 was a foray into this domain. GBM AGILE is a second step. Together they show how multiple hypotheses can be addressed in clinical trials. Such hypotheses include those regarding which patients respond to which therapy and how such hypotheses can be generated in a trial and confirmed in the same trial.

## Figures and Tables

**Table 1:** Posterior probabilities for the indicated possibilities cumulated to the end of the indicated year [47]. Antagonistic means Pravigard is numerically less effective than placebo. Synergistic means that the effect of Pravigard Pac is greater than the sum of the effects of single agents pravastatin and aspirin. The posterior probability that Pravigard Pac is effective, that is, superior to placebo, over the 5-year period is 0.9992.

Combination	Year 1	Year 2	Year 3	Year 4	Year 5
Antagonistic (effect is less than placebo)	0.0318	0.0072	0.0012	0.0002	0.0008
Positive, but less than additive	0.2050	0.1162	0.0444	0.0414	0.0660
Synergistic (greater than additive)	0.7632	0.8766	0.9544	0.9584	0.9332

**Table 3:** Bayesian characteristics of designs and analyses in Section 4 that have been conducted since 1995 or are still being conducted illustrate the use of and benefits of the Bayesian approach. Trials 1, 4, 5–7, 9, and 12–15 (shown in green shading) are for product registration (such as phase 3 drug trials) or for comparative effectiveness in the sense of influencing treatments used in clinical practice. Most of these characteristics are self-explanatory. “Efficient” means a smaller sample size, usually, than traditional trials that have comparable operating characteristics of type I error and statistical power.

Bayesian Characteristics	1	2	3	4	5	6	7	8	9	10	11	12	13	14	15
Adaptive	√	√	√	√	√		√	√	√	√	√	√	√	√	√
Efficient	√	√	√	√	√	√	√	√	√	√	√	√	√	√	√
Address many questions		√	√	√		√	√	√	√	√	√	√	√	√	√
Modeling/borrowing		√	√	√		√	√	√	√	√	√	√	√	√	√
Predictive probability	√	√	√		√		√	√	√	√	√		√	√	√
References	[24]	[36,37,38]	[40,41]	[9,42]	[44]	[46,47]	[48,52]	[5,6,7,55]	[66,67]	[69,70,71,72]	[73,74]	[5,6,7,79]	[84,85,86]	[89]	[94,95]

## Data Availability

This article describes many different trials with different sponsors. The data from a particular trial may or may not be available from its sponsor. Interested parties should contact the author at don@berryconsultants.net. He will inform the interested party of the disposition of the data and the ownership of the data for any particular trial.
